# The nucleoid as a smart polymer

**DOI:** 10.3389/fmicb.2015.00424

**Published:** 2015-05-08

**Authors:** Vittore F. Scolari, Bianca Sclavi, Marco Cosentino Lagomarsino

**Affiliations:** ^1^Computational and Quantitative Biology, Sorbonne Universités, UPMC Univ Paris 06, UMR 7238Paris, France; ^2^Centre National de la Recherche Scientifique, LBPA, UMR 8113, ENS CachanCachan, France; ^3^Centre National de la Recherche Scientifique, UMR 7238Paris, France

**Keywords:** genome organization, bacterial nucleoid, nucleoid-associated proteins, supercoiling, smart-polymers, polymer sensors

Science has a close but very complex relationship with technology (Latour, 1987). A simple phenomenon is that technology enables science by offering tools that provide new data or new kinds of data. In other cases, aspects or views of the empirical world may remain invisible until technology builds something that unveils them to the eyes of the scientific community. On a deeper level, building something may be a form of understanding. For example “complex networks” became prominent in all sectors of science in the late 1990s, at the time that the Internet became a common tool for research and for society at large. Before then, networks had been restricted for decades to smaller niches. This change was accompanied by a thrust of high throughput technologies to collect new data, but arguably many of the “network” data had already been available for many years.

On a smaller scale, we want to suggest here that so called “smart polymers” (Galaev and Mattiasson, 1999; Kumar et al., 2007) could be a promising technological metaphor for the behavior of the bacterial nucleoid. We want to explore the analogy with the similarly “intelligent” behavior shaped into bacterial nucleoids by natural selection.

But first, what is a smart polymer, and what does it do? In soft-matter physics, “smart,” or “stimulus-responsive,” polymers are technological polymer systems designed to effect a variety of responsive behaviors to external stimuli (Figure 1). Smart polymers respond to the environment they are in. They are engineered to be sensitive to a number of factors, such as solvency, temperature, humidity, pH, light, electrical and magnetic field, and to effect mechanical and chemical changes (Galaev and Mattiasson, 1999; Kumar et al., 2007; Chen and Chang, 2014). They can be realized as linear free chains in solution, or as surface-grafted brushes or gels. Usually, response to stimuli is achieved through the addition of specific reactive functional groups and side chains, or by the use of graft-and-block copolymers (two different polymers grafted together) with different chemical properties (e.g., hydrophyly). Effective smart polymers typically undergo large changes (e.g., conformational transitions) in response to just small changes in the environment (e.g., pH, temperature, ionic strength). One way to achieve this behavior is through the introduction of “pre-programmed” phase transitions. For example, the polymer undergoes a reversible collapse after an external stimulus is applied. The reversibility of this change may also be an important property, allowing to detect changes in both directions. To fix the ideas, a prevalent use for smart polymers is targeted drug delivery. A smart-polymer system may control the release of drugs until the desired target is reached, and it is sensed by either a chemical or physical response triggering the release of the drug by “uncaging” it. For example, a polymer site-specific conjugation to specific amino acid sites may induce a trigger in the concentration of a targeted protein (Hoffman et al., 2000). It is then evident that the bacterial nucleoid can be seen as a smart polymer (Dillon and Dorman, 2010; Muskhelishvili et al., 2010; Benza et al., 2012; Kleckner et al., 2014). Its degree of compaction and conformation are modulated by the cell's growth conditions and in response to specific external cues (Figure 1). It is a complex system made of a long DNA polymer associated with RNA and proteins that may play at least two roles: adapt the shape of the nucleoid through both specific and non-specific DNA binding, and change the physical properties of DNA through dynamic changes in DNA topology.

**Figure 1:**
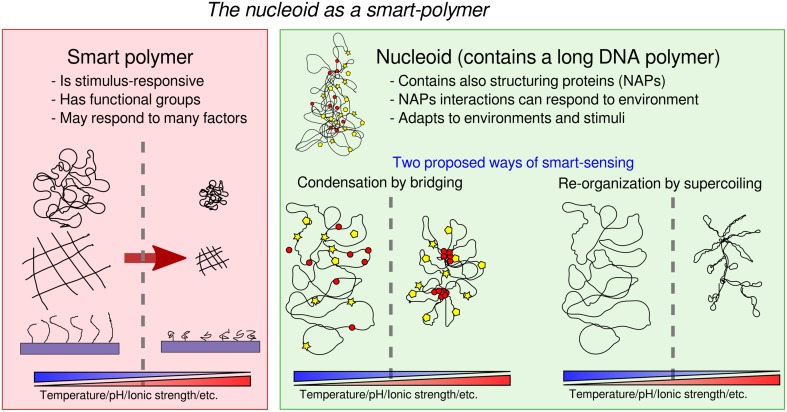
**“Smart-polymer” functionality of the nucleoid**.

First, the abundant nucleoid associated proteins (“NAPs,” e.g., focusing on *E. coli*, Dps, Fis, H-NS, IHF, HU, and the condensin MukBEF), can act as “functional groups” (Luijsterburg et al., 2006; Dillon and Dorman, 2010; Ohniwa et al., 2011) to plastically modify the genome conformation. Of particular interest are NAP-mediated bridging interactions (Wiggins et al., 2009) (e.g., from Fis, H-NS, and MukBEF in *E. coli*), which can thus act as “functional groups” in the nucleoid. In particular, H-NS is known respond to temperature, salt concentration and pH (La Teana et al., 1991; Atlung and Ingmer, 1997; Amit et al., 2003; Dorman, 2004; Ono et al., 2005; Stella et al., 2006). and Fis has been implicated in adaptation to favorable growth conditions and quorum sensing (Lenz and Bassler, 2007). Additionally, some NAPs may operate both as monomers and as oligomers, introducing the possibility of cooperativity in the formation of higher order complexes (Luijsterburg et al., 2006; Skoko et al., 2006; Lim et al., 2012). On theoretical grounds, looped domain formation offers the opportunity of producing a very rich phase behavior (Leibler, 1980; Borisov and Halperin, 1996, 1997; Kantor and Kardar, 1996; Camacho and Schanke, 1997), as exploited in recent models motivated by the study of the organization of chromatin (Junier et al., 2010; Barbieri et al., 2013; Brackley et al., 2013). Biologically, one can imagine that the collapse and swelling of selected genomic regions by bridging proteins may be tuned to be switch-like (Scolari and Cosentino Lagomarsino, 2015) in order to be differentially controlled by the cell. While the mechanisms has not yet been studied in detail, domain formation is well-documented in bacterial chromosomes (Espéli and Boccard, 2006; Espéli et al., 2008; Dame et al., 2011). Such mechanism may account for the observed correlation between the position of genetic loci along the chromosome and their position in the cell (Mercier et al., 2008; Wiggins et al., 2010), it may help the resolution of the identity of segregating sister chromosomes (Lesterlin et al., 2012; Junier et al., 2014), as well as play a role in explaining observed “abrupt” transitions in chromosome arrangements (Joshi et al., 2011; Fisher et al., 2013; Javer et al., 2014). Additionally, NAPs that do not bridge specifically such as Dps may also trigger switch-like collapse (Zimmerman, 2006), and NAPs that do not bridge but exhibit cooperative clustering may also affect the global nucleoid state by affecting key parameters such as effective stiffness (Luijsterburg et al., 2006).

Second, the action of specific DNA enzymes such as topoisomerases and gyrases changes the polymer's mechanical properties through changes in DNA topology. Nucleoids are composed of topologically unlinked dynamic domain structures, forming plectonemes and toroids (Trun and Marko, 1998). Torsional constraints can be generated by active processes, such as DNA replication and transcription (Le et al., 2013), and stabilized by bridging NAPs, such as Fis and H-NS (Schneider et al., 2001). Together, supercoiling and nucleoid organization can affect gene expression (Breier and Cozzarelli, 2004; Postow et al., 2004; Travers and Muskhelishvili, 2005; Blot et al., 2006; Dillon and Dorman, 2010) and, in turn, expression of specific regulators may affect the concentration or the activity of the genes setting nucleoid conformation resulting in feedback loops that can lead to more robust nucleoid conformations. Also, NAPs and supercoiling regulation by enzymes may interact in complex ways (Dorman, 2013a). Clearly, such an object has higher computational power than any current technological smart polymer, because it is also able to control the elements leading to its self-organization, which may inspire new technology. The coexistence of two parallel mechanisms of regulation through polymer organization (mainly supercoiling and growth) and through conventional protein binding may be an important feature of the nucleoid. A series of studies on *E. coli* investigated the interactions between these mechanisms arguing the presence of two different codes overlapped at different at levels on DNA, and possibly evolving at different time-scales, carrying, respectively, a “digital” and an “analog” information (Sobetzko et al., 2012; Dorman, 2013b; Muskhelishvili and Travers, 2013; Sobetzko et al., 2013). Finally this system is able to rapidly evolve in response to adaptation to recurring changes (Crozat et al., 2010), possibly improving the efficiency and the speed of the programmed conformational changes.

We propose that this technological parallel could also be useful in the reverse direction, to reframe the current biological knowledge in a physical perspective. Indeed, the smart polymer analogy does not by itself add new knowledge to the long list of biological information already acquired on the nucleoid. However, it may help us putting the same knowledge in a different perspective, and treat the same information in more precise and quantitative ways using the tools of soft-matter physics. This may lead to defining new questions, and ultimately to reaching new knowledge. For example, new biomimetic “constructive” approaches using purified DNA and NAPs may be defined to explore the resulting phase diagram in a controlled fashion (Maurer et al., 2009; Pelletier et al., 2012; Thacker et al., 2014), and to achieve a physical understanding of how robustness and response to changes are encoded in such structures.

## Conflict of interest statement

The authors declare that the research was conducted in the absence of any commercial or financial relationships that could be construed as a potential conflict of interest.
